# *Tmprss12* is required for sperm motility and uterotubal junction migration in mice^†^

**DOI:** 10.1093/biolre/ioaa060

**Published:** 2020-04-25

**Authors:** Tamara Larasati, Taichi Noda, Yoshitaka Fujihara, Keisuke Shimada, Tomohiro Tobita, Zhifeng Yu, Martin M Matzuk, Masahito Ikawa

**Affiliations:** 1 Department of Experimental Genome Research, Research Institute for Microbial Diseases, Osaka University, Suita, Osaka, Japan; 2 Graduate School of Medicine, Osaka University, Suita, Osaka, Japan; 3 Center for Drug Discovery, Baylor College of Medicine, Houston, TX, USA; 4 Department of Pathology and Immunology, Baylor College of Medicine, Houston, TX, USA; 5 The Institute of Medical Science, The University of Tokyo, Tokyo, Japan

**Keywords:** CRISPR/Cas9, knockout mice, male infertility, sperm motility, UTJ migration, fallopian tube, genome editing

## Abstract

Spermatozoa are produced in the testis but gain their fertilizing ability during epididymal migration. This necessary step in sperm maturation includes posttranslational modification of sperm membrane proteins that includes protein processing by proteases. However, the molecular mechanism underpinning this epididymal sperm maturation remains unknown. In this study, we focused on transmembrane serine protease 12 (*Tmprss12*). Based on multi-tissue expression analysis by PCR, *Tmprss12* was specifically expressed in the testis, and its expression started on day 10 postpartum, corresponding to the stage of zygotene spermatocytes. TMPRSS12 was detected in the acrosomal region of spermatozoa by immunostaining. To reveal the physiological function of TMPRSS12, we generated two knockout (KO) mouse lines using the CRISPR/Cas9 system. Both indel and large deletion lines were male sterile showing that TMPRSS12 is essential for male fertility. Although KO males exhibited normal spermatogenesis and sperm morphology, ejaculated spermatozoa failed to migrate from the uterus to the oviduct. Further analysis revealed that a disintegrin and metalloprotease 3 (ADAM3), an essential protein on the sperm membrane surface that is required for sperm migration, was disrupted in KO spermatozoa. Moreover, we found that KO spermatozoa showed reduced sperm motility via computer-assisted sperm analysis, resulting in a low fertilization rate in vitro. Taken together, these data indicate that TMPRSS12 has dual functions in regulating sperm motility and ADAM3-related sperm migration to the oviduct. Because *Tmprss12* is conserved among mammals, including humans, our results may explain some genetic cases of idiopathic male infertility, and TMPRSS12 and its downstream cascade may be novel targets for contraception.

## Teaser

TMPRSS12 has dual functions for uterotubal junction migration ability by affecting ADAM3 processing and sperm motility.

## Introduction

In mammals, sperm morphology is completed in the testis, but testicular spermatozoa are not yet competent to fertilize oocytes. Fertilizing ability (such as motility, capacitation, acrosome reaction, and sperm–oocyte fusion capabilities) is acquired during epididymal transit [1]. During sperm maturation in the epididymis, it is well-known that not only translational modification of proteins synthesized in spermatogenesis but the incorporation of new factors from the epididymal epithelium are also necessary. Finally, mature spermatozoa are stored in the cauda region of the epididymis until ejaculation.

During sperm maturation, immature forms of sperm surface proteins synthesized in the testes are subjected to posttranslational modification during epididymal transit. One example of this phenomenon is a disintegrin and metalloprotease 3 (ADAM3). The immature form of ADAM3 undergoes proteolytic processing in the epididymis, resulting in a decrease in size to form the mature ADAM3 that localizes on the membrane surface of the sperm head. ADAM3 maturation begins in the endoplasmic reticulum (ER) of testicular germ cells (TGCs), and ER chaperone proteins, such as calmegin (CLGN) [2], calsperin (CALR3) [3], and protein disulfide isomerase-like protein of the testis (PDILT) [4], are involved in proper ADAM3 protein folding. It was predicted that ADAM3 is stabilized by ADAMs oligomeric complexes in ER–Golgi intermediate compartment and Golgi. Once presented on the sperm surface, ADAM3 proteins undergo further modifications by GPIases and other proteases [5]. *Adam3* KO male mice are infertile due to failure of sperm to migrate through the female uterotubal junction (UTJ), preventing them from reaching the oocytes in the oviduct. The spermatozoa of these mice also fail to bind properly to the oocyte zona pellucida [6].

Serine proteases make up the major class of proteases involved in protein modification during sperm maturation [7]. Specifically, disruption of *Pcsk4*, an active serine protease detected exclusively in spermatocytes and spermatids, results in severe subfertility due to defective sperm capacitation; the KO spermatozoa exhibit accelerated capacitation, resulting in less-efficient sperm–oocyte binding [8, 9]. Although PRSS37 is an inactive protease, loss of *Prss37* causes male infertility in mice because the KO spermatozoa fail to migrate to the oviduct; ADAM3 vanishes in *Prss37* KO spermatozoa due to problems in ADAM3 modification [10].

As explained above, soluble serine proteases have been widely studied for their roles in reproduction. In this study, we focused on a transmembrane serine protease (TMPRSS) as it had the potential to modify other membrane proteins in the testes and spermatozoa. TMPRSS is a subfamily of transmembrane type serine protease. To date, 19 TMPRSSs have been identified in mice and 17 have human homologs [11]. TMPRSS members, except TMPRSS12, are ubiquitously expressed, and they are widely studied for their roles in virus spread (TMPRSS2) [12] and cancer progression (TMPRSS4 and HEPSIN) [13, 14]. *Tmprss12* is expressed specifically in the testis based on in silico analysis. In human, *TMPRSS12* is predominantly expressed in the testis, and TMPRSS12 localizes to the cell membrane of spermatocytes and spermatids [15]. However, the role of *Tmprss12* in male reproduction has not yet reported. Here, we generated *Tmprss12* KO mice using the CRISPR/Cas9 system and showed that *Tmprss12* contributes to sperm migration to the oviduct and sperm motility.

## Materials and methods

### Animal ethics

Wild-type (WT) B6D2F1, C57BL/6J, and ICR mice were purchased from CLEA Japan, Inc. (Tokyo, Japan) or Japan SLC, Inc. (Shizuoka, Japan). They were acclimated to a 12-h light/12-h dark cycle, and could freely drink and eat anytime. All samples were collected after euthanasia. All experiments involving animals were approved by the Institutional Animal Care and Use Committees of Osaka University (Osaka, Japan) (#Biken-AP-H25-02 and #Biken-AP-H30-01) and were conducted in compliance with the guidelines and regulations for animal experimentation of both institutions.

### Antibodies

Rabbit polyclonal antibody was produced by immunization with mouse TMPRSS12 polypeptide (residues 111–130: CTKEARDPLKWRAVMGTNDL, Sigma-Aldrich, St. Louis, MO). The TMPRSS12 antibody was purified using the TMPRSS12 polypeptide and SulfoLink coupling resin (Thermo Fisher Scientific, Waltham, MA). Other antibodies are as described previously, IZUMO1 (KS64–125) [3], or purchased: ADAM3 (sc-365288, Santa Cruz Biotechnology, Santa Cruz, CA) and GAPDH (14C10, Cell Signaling Technology, Beverly, MA).

### In silico analysis

Conservation of TMPRSS12 among species was examined using TreeFam (http://www.treefam.org/). TreeFam is a database indicating the phylogenetic trees predicted from animal genomes. The transmembrane region of TMPRSS12 was predicted using Phyre2 (http://www.sbg.bio.ic.ac.uk/∼phyre2/). Expression of *Tmprss* family genes was examined using UniGene (https://www.ncbi.nlm.nih.gov/unigene). Mouse *Tmprss12* genome sequence was from Ensemble (http://asia.ensembl.org/). Messenger RNA and amino acid sequence were from NCBI (https://www.ncbi.nlm.nih.gov/gene/): mouse *Tmprss12* (mRNA, NM_183109; protein, NP_898932) and human *TMPRSS12* (mRNA, NM_182559; protein, NP_872365).

### Reverse transcription polymerase chain reaction

Reverse transcription polymerase chain reaction (RT-PCR) analysis was performed as described previously [16]. In brief, mouse samples (testis, epididymis, ovary, uterus, and eight non-reproductive tissue types [brain, heart, kidney, liver, lung, small intestine, spleen, and stomach]) were obtained from dissection of C57BL6/129SvEv mice. Human multi-tissues were obtained from the Human Tissue Acquisition and Pathology core service (Baylor College of Medicine, Houston, TX). Informed consent of these human tissues was obtained. The primers used are as follows:

Mouse *Tmprss12*: 5′-CACAGCGTGTTTTATAAGCGG-3′ and 5′-ATGTCCGTAACTGGTGATTCC-3′ (multi-tissue, 30 cycles; postnatal mouse testis, 35 cycles).

Mouse *Hprt*: 5′-TGGATATGCCCTTGACTATAATGAG-3′ and 5′-TGGCAACATCAACAGGACTC-3′ (multi-tissue, 35 cycles; postnatal mouse testis, 35 cycles).

Human *TMPRSS12*: 5′-GGAGCTTTTGATACTTGCAGG-3′ and 5′-GGATGGCCCAATATAGACACC-3′ (30 cycles).

Human GAPDH: 5′-AATCCCATCACCATCTTCCAG-3′ and 5′-ATGACCCTTTTGGCTCCC-3′ (30 cycles)

### gRNA design

Potential gRNA and PAM sites were found using the online source CRISPRdirect [17]. Guide RNAs with fewer off-target sites were chosen.

### Plasmid and oligonucleotide preparation

Plasmids expressing hCas9 and gRNA were prepared by ligating oligonucleotides into the *Bbs*I site of pX330 [18] (http://www.addgene.org/42230/). The pCAG-EGxxFP target plasmid was prepared as previously described [19] (http://www.addgene.org/50716/). The primers used for the pCAG-EGxxFP construction are as follows: 5′-CCAGGTTGTCTCAGCTCTGTGATTGC-3′ (plus *Nhe*I site) and 5′-CAAGACCCTCACACAGACACATGCAG-3′ (plus *Sal*I site). The gRNA target sequence for the 8 bp deletion was 5′-GAAGGGTCTCGGATTATAGG-3′ (exon 2). The gRNA target sequences for large deletion were the sequence used for 8 bp deletion and 5′-ATCCTACTAGGAGTAACATA-3′ (exon 5).

### Pronuclear injection

Hormone-treated B6D2F1 female mice were mated with B6D2F1 males, and fertilized eggs were collected from the oviduct. The pX330 plasmid containing the target gRNA sequence was injected into one of the pronuclei of a fertilized egg at 5 ng/μl. The injected eggs were cultivated in potassium simplex optimization medium (KSOM) [20] overnight, and the two-cell stage embryos were transferred into the oviducts of pseudopregnant ICR females at 0.5 days after mating with vasectomized males. The pups obtained were genotyped by PCR and then subsequently confirmed by Sanger sequencing. After genotype validation, heterozygous F0 mice underwent serial mating to generate homozygous mutant offspring.

### Electroporation

Hormone-treated B6D2F1 female mice were mated with B6D2F1 males, and fertilized eggs were collected from the oviduct. Ordered crRNAs (Sigma-Aldrich) and tracrRNA (#TRACRRNA05N-5NMOL, Sigma-Aldrich) were annealed, mixed with CAS9 protein (#B25640, Thermo Fisher Scientific), and then were incubated at 37 °C for 5 min to make the gRNA/Cas9 ribonucleoprotein complex (final concentration: 40 ng/μl gRNA and 100 ng/μl CAS9). The obtained complex was electroporated into fertilized eggs using a superelectroporator NEPA21 (NEPA GENE, Chiba, Japan) (poring pulse, voltage, 225 V; pulse width, 2 ms; pulse interval, 50 ms; number of pulses, +4, attenuation 10%; transfer pulse, voltage, 20 V; pulse width, 50 ms; pulse interval, 50 ms; number of pulses, ±5, attenuation 40%). The eggs were cultivated in KSOM [20] overnight, and the two-cell stage embryos were transferred into the oviducts of pseudopregnant ICR females. The pups obtained were genotyped by PCR and then subsequently confirmed by Sanger sequencing.

### PCR for genotyping of subsequent generations

PCR analysis of genomic DNA was performed for *Tmprss12^d8/d8^* mutant mice using primer set Fw-1: 5′-GAACATGGGACTTAGCTAGTG-3′ and Rv-1: 5′-CCTCTTTTGTGCAGTGAGCAGC-3′. Direct Sanger sequencing of PCR products was then performed. Two sets of primers were used to genotype the *Tmprss12^del/del^* mutant mice: one that amplified 617 bp in exon 3 to check the WT allele (Fw-2: 5′-GATCCTCTGAAGTGGAGAGCAGTGATG-3′ and Rv-2: 5′-GGGTTCCTACTTGTGCTTGCGCAA-3′) and one that only amplified 3,278 bp if the large deletion allele is present (Fw-1: 5′-GAACATGGGACTTAGCTAGTG-3′ and Rv-3: 5′-CAAGACCCTCACACAGACACATGCAG-3′). The amplification conditions for the subsequent PCR were 1 min at 94 °C, followed by 30 or 35 cycles of 94 °C for 30 s, 65 °C for 30 s and 72 °C for 30 s, with a final 7-min extension at 72 °C.

### Protein extraction

TGCs were squeezed from testicular tubules under the microscope, minced with razor in PBS, and then filtrated using 59 μm nylon mesh filter. Spermatozoa were squeezed from the cauda epididymis and vas deferens into PBS. After centrifugation at 2000 rpm for 5 min and PBS removal, TGC or spermatozoa were homogenized in 1 × lysis buffer (ab152163, Abcam, Cambridge, MA) containing protease inhibitor cocktail (Nacalai Tesque, Kyoto, Japan) for overnight at 4 °C on rotator and then centrifuged at 2000 rpm for 5 min with the supernatants before being collected.

### Immunoblot analysis

Immunoblot analysis was performed as described previously [2]. Protein lysates were resolved by SDS/PAGE under reducing condition and transferred to PVDF membranes. After blocking with 10% skim milk (#232100, Nacalai Tesque) diluted in TBST (Tris-buffered saline, 0.1% Tween 20), blots were incubated with primary antibodies overnight at 4 °C and then incubated with secondary antibodies conjugated to horseradish peroxidase (1:10,000) (Jackson ImmunoResearch, West Grove, PA) for 1 h at RT. Antibodies used are anti-TMPRSS12 1:500, anti-ADAM3 1:1000, and anti-GAPDH 1:1000. Antibodies were diluted in blocking solution. Detection was performed using Chemi-Lumi One Super (Nacalai Tesque).

### Immunofluorescence analysis

For spermatozoa, after 3 h incubation in TYH, samples were diluted in PBS, spread onto microscope slides, and were incubated at 37 °C until dry. The samples were fixed with 4% paraformaldehyde in PBS for 10 min. After three 5-min washes with PBS, the slides were treated with 1% Triton X-100 for 10 min followed with three 5-min washes with PBS. Then, the slides were blocked with 10% goat serum diluted in PBS for 1 h at RT. The slides were incubated with anti-TMPRSS12 (1:50) and anti-IZUMO1 (1:200) diluted in blocking solution overnight at 4°C and then washed with PBS 3 times for 5 min each. After incubation with secondary antibody at RT for 1 h, slides were washed with PBS for 5 min, incubated with Hoechst 33342 for 5 min, washed with PBS, and then mounted. Slides were viewed with an Olympus BX50 microscope (Tokyo, Japan).

### Fertility testing of male mice

Upon sexual maturation, three *Tmprss12* KO male mice were caged individually with three 8-week-old B6D2F1 female mice for 2 months, and plugs were checked every morning. For controls, three WT mice were used. The number of pups was counted on the day of birth. After the mating period, male mice were removed from the cages, and WT females were kept for another 3 weeks to allow them to deliver their final litters. The average litter size for each mouse line was calculated by dividing the total number of pups with the number of litters. Frozen spermatozoa from *Tmprss12^d8/wt^* males (B6D2-Tmprss12 < em1Osb>, RBRC#10125, CARD#2598) and *Tmprss12^del/wt^* males (B6D2-Tmprss12 < em4Osb>, RBRC#11014, CARD#2921) will be available through RIKEN BRC (http://en.brc.riken.jp/index.shtml) and CARD R-BASE (http://cardb.cc.kumamoto-u.ac.jp/transgenic/).

### Testis and epididymis histology

Males were sacrificed by cervical dislocation. Testis and epididymis were fixed in 4% paraformaldehyde in PBS and were processed for plastic sectioning using a Technovit 8100 instrument (Kulzer, Wehrheim, Germany) according to the manufacturer’s instruction. Briefly, fixed testes were washed in PBS at 4 °C for 1 h, dehydrated in acetone at 4 °C for 1 h, infiltrated with in mixed solution of Technovit 8100 basic solution and hardener 1 (1.5 mL of basic solution plus 9 mg of hardener 1 per sample) at 4 °C for 2–6 h, and then embedded after adding 50 μl of hardener 2. For analysis of mouse testes, 5 μm sections were treated with 1% periodic acid for 10 min, followed by treatment with Schiff reagent (#193-08445, FUJIFILM Wako Pure Chemical, Osaka, Japan) for 20 min. The sections were stained with Mayer hematoxylin (#131-09665, FUJIFILM Wako Pure Chemical) solution prior to imaging and observed under microscope. For analysis of mouse epididymis, 5 μm sections were treated with Mayer hematoxylin solution for 3 min, then were stained with Eosin solution (1% Eosin Y solution [#051-06515, FUJIFILM Wako Pure Chemical] mixed with 80% ethanol in 1:2 ratio), and then added 0.5% of total volume of acetic acid for 2 min. Stained sections were then observed under microscope.

### Sperm morphology

Cauda epididymal spermatozoa were dispersed in TYH medium [21, 22] and observed under a phase contrast microscope (Olympus BX50 microscope) to assess morphology and motility.

### Ultrastructural analysis of spermatozoa using scanning electron microscopy

Cauda epididymal spermatozoa were incubated in TYH medium to disperse. Spermatozoa were collected into 2.0 ml tube and washed with 0.1 M phosphate buffer (pH 7.4). Spermatozoa were mounted on cover slips and fixed with 1% glutaraldehyde in 0.1 M phosphate buffer on ice. After washing, the specimens were post-fixed with 1% osmium tetroxide in 0.1 M phosphate buffer containing 1% potassium ferrocyanide and conductive-stained with 1% tannic acid solution and 1% osmium tetroxide solution. The specimens were dehydrated in graded series of ethanol and then critical point dried using a Samdri-PVT-3D system (Tousimis, Rockland, MD). The specimens were coated with osmium tetroxide using an osmium coater HPC-30 W (Vacuum Device, Ibaraki, Japan). Electron micrographs were captured with S-4800 field emission scanning electron microscope (Hitachi, Tokyo, Japan).

### Ultrastructural analysis of spermatozoa using transmission electron analysis

Sperm samples were prepared for transmission electron microscopic analysis as previously described [23].

### Sperm migration assay

Sperm migration assay was performed as described previously [6]. In brief, hormone-treated females were caged together with test males 12 h after hCG injection. Once plug formation was confirmed, the male was separated from the female. About 4 h after copulation, oviducts were excised, together with the connective part of the uterus. Oviducts were carefully separated from the uterine horns and straightened out, then were transferred to slides as whole mounts, covered with coverslips, and examined with Olympus BX50 and Keyence BZ-8000 (Osaka, Japan) microscopes.

### Sperm–ZP binding assay

Sperm binding ability to zona pellucida was tested as described previously [2]. In brief, the cumulus cell layer of oocytes from female B6D2F1 mice (8-week-old) was removed by treatment with bovine testicular hyaluronidase (175 U/ml; Merck) for 5 min. Cumulus-free eggs were then placed in TYH medium. An aliquot of capacitated sperm (2 x 10^5^ spermatozoa/ml) was inseminated, and the mixture was incubated for 30 min at 37 °C under 5% CO_2_ in air. Oocytes and fertilized eggs were fixed with 0.2% glutaraldehyde, and the bound sperm were observed with an IX-73 fluorescent microscope (Olympus).

### In vitro fertilization

In vitro fertilization was performed as described previously [4]. In brief, eggs collected from hormone-treated females 14 h after hCG injection were placed in 100 μl of (a) TYH medium [21, 22] or (b) CARD medium (Kyudo, Saga, Japan). After 2 h of sperm incubation, capacitated epididymal sperm were added to the drop containing eggs at a final concentration of 2 x 10^5^ spermatozoa/ml. After 2 h of coincubation, inseminated eggs in CARD medium were moved to KSOM to prevent polyspermy. After 8 h of coincubation, the formation of pronuclei was observed under a Hoffman modulation contrast microscope. Two-cell embryos were counted at around 32 h after coincubation.

### Sperm velocity analysis

Cauda epididymal spermatozoa were suspended in TYH medium [21, 22]. Sperm velocity was measured using the CEROS II sperm analysis system (Hamilton Thorne Biosciences, Beverly, MA) at 10 min and 2 h after incubation. More than 200 spermatozoa were analyzed for each male.

### Statistics

Statistical analyses were performed using Student *t*-test (for Supplementary Figure S3D, F and G), Welch *t*-test (for Figure 4D; Supplementary Figure S3F), Mann–Whitney U test (for Supplementary Figure S3E and F), Tukey–Kramer test (for Figure 4C), and Steel–Dwass test (for Figure 4A–C; Supplementary Figure S4B) after examining normal distribution and variance. Error bars shown as standard deviation (s.d.). All statistical analyses were conducted using Microsoft Office Excel (Microsoft Corporation, Redmond, WA) with the add-in software Statcel3 (OMS, Saitama, Japan).

## Results

### Expression of *TMPRSS12* in the testis and spermatozoa

*Tmprss12* is evolutionarily conserved in many vertebrate species including birds, frogs, lizards, and mammals (Supplementary Figure S1A). Sequence alignment of mouse and human TMPRSS12 showed 64% identity and 93% similarity (Supplementary Figure S1B). The protein is a type 1 transmembrane protein, the precursor contains a signal peptide, the transmembrane region was detected at the C-terminus, and a trypsin-like serine protease domain is located in the extracellular region by in silico analysis (Supplementary Figure S1C), although the enzyme activity is unclear.

**Figure 1:**
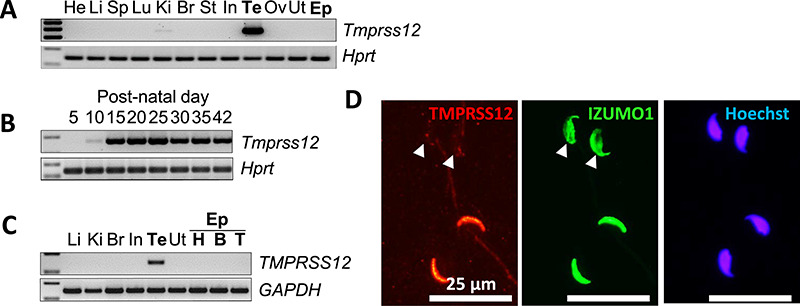
Characterization of mouse *Tmprss12*. (A) Expression of mouse *Tmprss12* was examined by RT-PCR using RNA isolated from various organs. *Tmprss12* is testis-enriched. The *Hprt* gene was used as an expression control. He, heart; Li, liver; Sp, spleen; Lu, lung; Ki, kidney; Br, brain; St, stomach; In, intestine; Te, testis; Ov, ovary; Ut, uterus; Ep, epididymis. (B) Expression of mouse *Tmprss12* in postnatal days in the testis was examined by RT-PCR. *Tmprss12* begins expression at postnatal day 10. The *Hprt* gene was used as an expression control. (C) Expression of human *TMPRSS12* was examined by RT-PCR using RNA isolated from various organs. *TMPRSS12* is testis-specific. The *GAPDH* gene was used as an expression control. H, head (caput); B, body (corpus); T, tail (cauda). (D) Immunofluorescence analysis of spermatozoa from wild-type (WT) mice labeled with antibodies against TMPRSS12 (red) and IZUMO1 (green). Fluorescence was seen in the sperm head and reduced in acrosome-reacted spermatozoa (▲). Nuclei were stained with Hoechst 33342 (blue). Scale bars: 25 μm.

We conducted multi-tissue gene expression analysis by RT-PCR, and *Tmprss12* was abundantly expressed in mouse testis (Figure 1A). From RT-PCR using mouse testis from early post-natal days, we found that *Tmprss12* expression began at postnatal day 10, which corresponds to the stage of zygotene spermatocytes [24] (Figure 1B). Also, human *TMPRSS12* was specifically detected in testis (Figure 1C). Immunostaining of cauda epididymal spermatozoa revealed that TMPRSS12 localized to the sperm acrosomal region and the signal decreased after the acrosome reaction (Figure 1D**)**.

### Male fecundity of *Tmprss12* KO mice

**Figure 2:**
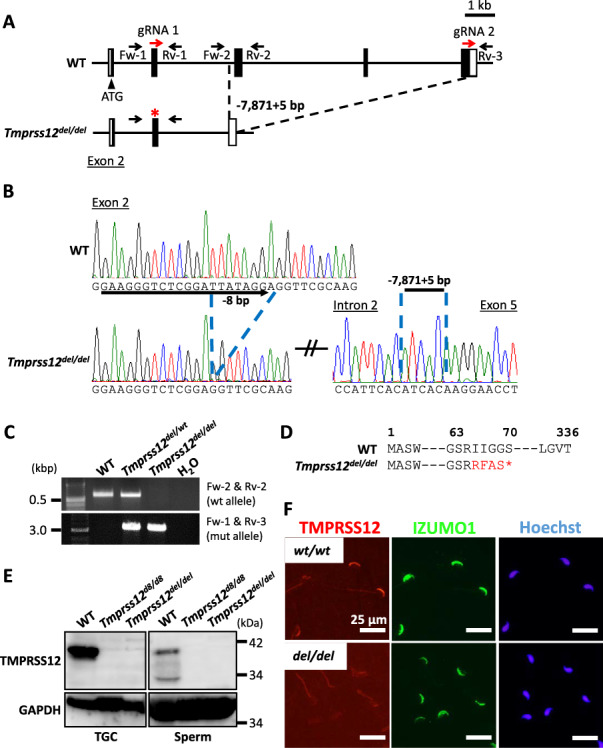
Generation of *Tmprss12^del/del^* mice using CRISPR/Cas9. (A) Structure of *Tmprss12* gene and CRISPR/Cas9 targeting scheme in exon 2 and 5. Asterisk (^*^) indicates the 8 bp deletion. Black boxes show the coding region. (B) Wave pattern sequence of *Tmprss12* in WT and *Tmprss12^del/del^* mice. (C) Genotyping of *Tmprss12^del/del^* mice using two primer sets that amplify the WT allele or the large deletion allele. (D) Amino acid translation in *Tmprss12^del/del^* mice. The large deletion in *Tmprss12^del/del^* mice causes a premature stop codon at amino acid 70. (E) Protein expression of TMPRSS12 in testis and cauda epididymal spermatozoa from WT, *Tmprss12^d8/d8^*, and *Tmprss12^del/del^* mice. GAPDH was used as a loading control. TGC: testicular germ cells. (F) Immunofluorescence analysis of spermatozoa from *Tmprss12 ^del/del^* mice labeled with antibodies against TMPRSS12 (red). Fluorescence observed in the WT sperm head disappears in spermatozoa from *Tmprss12^del/del^* mice. Nuclei were stained with Hoechst 33342 (blue). Scale bars: 25 μm.

To reveal the physiological function of *Tmprss12*, we generated two mutant mouse lines of *Tmprss12* using CRISPR/Cas9. One guide RNA (gRNA1) was designed at exon 2, downstream of the first methionine (Supplementary Figure S2A). By injecting gRNA1/Cas9 expressing plasmid into zygotes, two out of six founder generation (F0) mice with the mutant allele were obtained. Disruption of eight nucleotides in exon 2 in the next generation from F0 mutants was confirmed by direct Sanger sequencing (Supplementary Figure S2B). Moreover, to omit the possibility that translated products from aberrant alternative splicing mask the phenotype, we designed another gRNA (gRNA2) at exon 5 and obtained F0 deletion mutants by introducing gRNA1, gRNA2, and CAS9 enzyme into zygotes (1 mutant/12 delivered pups) (Figure 2A). This mouse line has the mutant allele with disrupted eight nucleotides in exon 2, identical to the indel mutant, with large deletion from intron 2 to exon 5 (*Tmprss12^–8,-7871 + 5/−8,-7871 + 5^,* subsequently to referred to as *Tmprss12^del/del^*) (Figure 2B and C). These deletions in both mutant lines cause frameshift mutations with a premature stop codon at the 70th amino acid (Figure 2D; Supplementary Figure S2C).

The KO mice obtained by heterozygous F1 intercross were able to mature without showing overt abnormalities. To determine the success of the KO at ablating the protein, we conducted a western blot analysis of TMPRSS12. An approximately 39 kDa band was strongly detected in WT; however, the band was absent at the protein level in both KO TGCs and spermatozoa (Figure 2E). The TMPRSS12 protein in WT spermatozoa also appears to be partially processed to a smaller form (Figure 2E), the significance of which is unknown. The signal in the sperm head also disappears in the *Tmprss12^del/del^* mice, as confirmed by sperm immunostaining (Figure 2F). We mainly used *Tmprss12^del/del^* males for further analyses.

**Figure 3:**
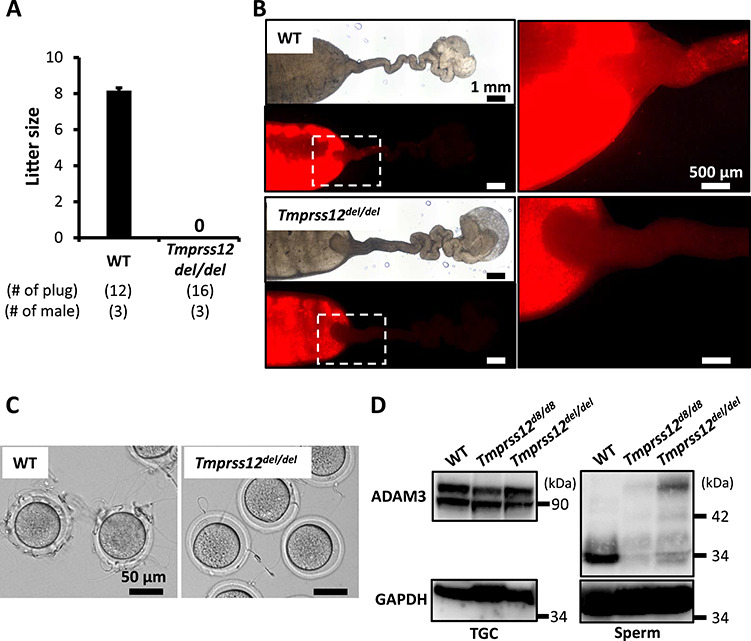
UTJ migration and ZP binding assay of spermatozoa from *Tmprss12^del/del^* mice. (A) Litter size (number of pup/litter) results of the mating test. WT and *Tmprss12^del/del^* mice were mated with 3 WT females per male. The litter size of WT males is 8.17 ± 0.14 pups/litter. (B) Uteri and oviducts from WT females mated with WT and *Tmprss12^del/del^* males carrying fluorescent protein-tagged spermatozoa. Spermatozoa from *Tmprss12^del/del^* mice fail to pass through the UTJ. Photographs were taken 4 h after coitus. Right figures are magnified images of the boxes indicated in the left figures. Scale bars: 1 mm (left) and 500 μm (right). (C) Observation of ZP binding in spermatozoa from WT and *Tmprss12^del/del^* mice. Spermatozoa from *Tmprss12^del/del^* mice have impaired ZP binding ability in vitro. Scale bars: 50 μm. (D) Immunoblot analysis of ADAM3 using TGC and cauda epididymal spermatozoa from *Tmprss12^d8/d8^* and *Tmprss12^del/del^* mice. The mature form of ADAM3 was disrupted in *Tmprss12* KO spermatozoa.

Next, we investigated the fecundity of KO male mice by caging individual WT or *Tmprss12* KO male with three WT females for 2 months. In contrast to control male mice, which were able to produce offspring continuously, *Tmprss12* KO males could not produce any offspring despite the formation of copulatory plugs (Figure 3A; Supplementary Figure S3A).

### Spermatogenesis in *Tmprss12* KO male mice

Since *Tmprss12* was abundantly expressed in the testis, to determine the cause of male sterility, we first characterized the spermatogenesis of KO male mice. *Tmprss12* KO testis showed no significant differences at morphology and weight (Supplementary Figure S4A and B). There were no defects found in the spermatogenesis of KO male mice by histological analysis (Supplementary Figure S4C). The spermatozoa were observed in caput and cauda epididymis of WT and KO males at a comparable level (Supplementary Figure S4D). Then, we collected KO spermatozoa from the cauda epididymis to observe morphology, but these sperm appeared normal (Supplementary Figure S5A). Furthermore, ultrastructure analysis using electron microscopy showed that KO spermatozoa had a normal sperm head shape, normal mitochondrial alignment in the midpiece, and normal microtubule structure of the midpiece and principal piece (Supplementary Figure S5B and C).

### *Tmprss12* KO sperm behavior in the female reproductive tract was abnormal

To observe sperm behavior in the female reproductive tract, we crossed KO mice with a transgenic mouse line in which the acrosome and midpiece regions of spermatozoa are transgenically labeled with green and red fluorescence, respectively [25]. When we observed the ejaculated spermatozoa through the wall of the female reproductive tract, WT spermatozoa could migrate from the uterus through the UTJ to the oviduct. However, although enough counts of KO spermatozoa were present inside the uterus and around the UTJ, these spermatozoa failed to migrate into the oviduct (Figure 3B; Supplementary Figure S3B). When we inseminated *Adam3* KO and *Tmprss12* KO spermatozoa with cumulus-free oocytes in vitro, *Tmprss12* KO spermatozoa rarely bound to ZP (Figure 3C; Supplementary Figure S3C).

ADAM3 is an essential sperm factor for sperm UTJ migration and ZP binding [6]. When we characterized ADAM3 protein levels and molecular weight in *Tmprss12* KO TGC and spermatozoa, the immature ADAM3 was detected in WT and KO TGC at a comparable level, but we barely observed mature ADAM3 in the KO spermatozoa (Figure 3D). From these results, we conclude that *Tmprss12* KO mice are sterile due to a sperm migration defect through the UTJ as the result of ADAM3 disruption.

### Fertilizing ability of *Tmprss12* KO spermatozoa in vitro

**Figure 4:**
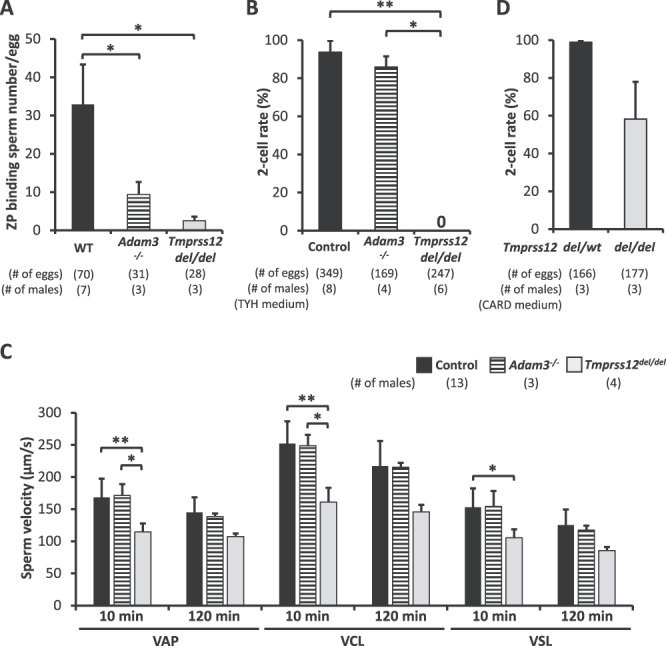
Fertilizing ability and motility analysis of spermatozoa from *Tmprss12 ^del/del^* mice. (A) Average numbers of ZP binding spermatozoa in vitro. The number of ZP binding spermatozoa in *Tmprss12^del/del^* mice (2.49 ± 1.07 spermatozoa), and *Adam3^−/−^* mice (9.40 ± 3.23 spermatozoa) were significantly reduced compared to that of WT mice (32.93 ± 10.43 spermatozoa). ^*^P < 0.05. (B) IVF rates in TYH using spermatozoa from control, *Adam3^−/−^*, and *Tmprss12^del/del^* mice. Average fertilization rates of control, spermatozoa from *Adam3^−/−^*, and *Tmprss12^del/del^* mice were 93.7 ± 6.0%, 85.9 ± 5.7%, and 0%, respectively. Spermatozoa from WT and *Tmprss12^del/wt^* mice were used as controls. ^*^P < 0.05, ^*^^*^P < 0.01. (C) Computer-assisted sperm analysis motility parameters; (left to right) average path velocity (VAP), curvilinear velocity (VCL), straight line velocity (VSL). Spermatozoa were incubated in capacitation medium (TYH) and motility measured at 10 min (uncapacitated) and 120 min (capacitated) to assess motility before and after capacitation. More than 200 spermatozoa were analyzed for each male. Spermatozoa from WT, *Tmprss12^d8/wt^*, and *Tmprss12^del/wt^* mice were used as controls. *Tmprss12^del/del^* KO sperm velocity decreased compared to those of controls and *Adam3* KO males. ^*^P < 0.05, ^*^^*^P < 0.01. (D) In vitro fertilization rates spermatozoa from *Tmprss12^del/wt^* and *Tmprss12^del/del^* mice in CARD medium. Average fertilization rates of spermatozoa from *Tmprss12^del/wt^* and *Tmprss12^del/del^* mice were 98.8 ± 1.0 and 58.2 ± 19.7, respectively.

As explained above, ADAM3 disruption may relate to other phenotypes of *Tmprss12* KO mice; hence for the next analyses, we compared the characteristics of *Tmprss12* KO and *Adam3* KO spermatozoa. First, ZP binding defect showed in Figure 3C and Supplementary Figure S3C was quantitatively analyzed by counting the number of sperm bound to the ZP of cumulus-free eggs. Similar to *Adam3* KO and in contrast to WT spermatozoa, *Tmprss12* KO spermatozoa showed significantly low ZP binding ability (Figure 4A; Supplementary Figure S3D).

*Adam3* KO spermatozoa are able to fertilize cumulus-intact oocytes in vitro as previously reported [4]. Surprisingly, when we inseminated spermatozoa with cumulus-intact oocytes, the fertilization rate of *Tmprss12* KO spermatozoa was severely reduced compared to that of WT and *Adam3* KO spermatozoa (Figure 4B; Supplementary Figure S3E). These data suggest a more severe defect in *Tmprss12* KO spermatozoa compared to *Adam3* KO spermatozoa.

Despite normal morphology, the inability of spermatozoa to fertilize oocytes can be caused by various factors, such as low sperm motility, acrosome reaction defect, and egg activation defects. First, we examined sperm motility using computer-assisted sperm analysis system. Compared to WT and *Adam3* KO spermatozoa, *Tmprss12* KO spermatozoa showed reduced motility in all parameters (Figure 4C; Supplementary Figure S3F). When the ZP was weakened using CARD medium, the fertilization rate of *Tmprss12* KO spermatozoa increased (Figure 4D; Supplementary Figure S3G). Since the fertilization rate was rescued by weakening ZP, we can exclude sperm capacitation and egg activation defects as the cause of the low IVF rate. These data imply that reduced sperm motility contributes to the low fertilization ability of *Tmprss12* KO spermatozoa in vitro.

## Discussion

In this study, we generated *Tmprss12* KO mice using the CRISPR/Cas9 system, and these KO mice were infertile primarily due to failure of spermatozoa to migrate through the UTJ. ADAM3 processing was disrupted in the KO spermatozoa, and KO spermatozoa also exhibited ZP binding defect, similar to ADAM3 KO spermatozoa. Previously, KO analyses of 15 genes and gene clusters (*Ace*, *Adam1a*, *Adam2*, *Calr3*, *Clgn*, *Cmtm2a/Cmtm2b*, *Cst family*, *Pate8–Pate10*, *Pdilt*, *Pmis2*, *Prss37*, *Prss55*, *Rnase10*, *Tex101*, and *Tpst2*) have revealed that disruption of ADAM3 processing is highly relevant to UTJ migration failure [26–29]. There are two possibilities of how TMPRSS12 impacts ADAM3. One is that TMPRSS12 processes ADAM3-related proteins in the testis, which then impacts ADAM3 maturation in the epididymis. Since ADAM3 protein is normal in KO TGCs and TMPRSS12 is localized in the sperm head, it is highly possible that TMPRSS12 processes ADAM3 in spermatozoa in either a direct or indirect manner. However, since the protease activity of TMPRSS12 is unclear, TMPRSS12 ability to cleave proteins and its direct and/or indirect interactions with ADAM3 should be examined in future work. Analysis of ADAM3-related protein localization in KO testes and spermatozoa may reveal the connection between TMPRSS12 and ADAM3.

It was previously reported that a very low amount of mature ADAM3 in spermatozoa of transgenic mice, in which ADAM3 was expressed using the testis-specific calmegin promoter, could rescue the *Adam3* KO phenotype [27]. In *Tmprss12* KO spermatozoa, we could still detect very small amounts of the mature form of ADAM3, suggesting that the remaining ADAM3 is not functional enough to rescue the phenotype. In parallel, *Tmprss12* KO sperm showed reduced sperm motility in addition to the ADAM3 KO phenotypes. These results suggest that the UTJ migration failure might not be solely due to ADAM3 disruption but could be due to the combined effects of ADAM3 disruption and motility defect.

Sperm motility is highly related to roles of sperm tail proteins [30], but ultrastructure analysis showed that KO sperm tail had normal microtubule structure and mitochondria alignment. As *Tmprss12* expression starts from the early stage of spermatogenesis and TMPRSS12 was predicted to function in the extracellular space, it is possible that TMPRSS12 also processes motility-related membrane proteins, such as the ion channels CATSPERs and SLO3 [31, 32], during spermatogenesis. However, we cannot rule out the possibility that TMPRSS12 impacts sperm motility-related proteins during epididymal maturation.

Chicken TMPRSS12 promotes avian metapneumovirus fusion to host cell by cleaving a viral envelope glycoprotein. Mutation of the catalytic triad of histidine, aspartic acid, and serine residues (HDS) results in an impaired capacity of chicken TMPRSS12 in cleaving the viral envelope glycoprotein [33]. Since the HDS triad of TMPRSS12 is conserved among mammals, TMPRSS12 might also hold function as an active protease in mouse and human.

In conclusion, our study demonstrates that *Tmprss12* is essential for male fertility in mice by impacting ADAM3 maturation and sperm motility. Further work is required to elucidate the mechanism of action of TMPRSS12 in testes and spermatozoa. Studies on the protease features of TMPRSS12, such as activity and recognition motifs, as well as proteomic analysis, are needed to identify the interacting substrates of TMPRSS12. Our discovery that *Tmprss12* plays dual functions in fertility can be useful in explaining idiopathic male infertility, and since TMPRSS12 is conserved among mammals, including human, TMPRSS12 may be a novel target for the identification of male contraceptive molecules.

## Supplementary Material

supplementary_figures_ioaa060
