# Supplementary material for: Tmprss12 is required for sperm motility and uterotubal junction migration in mice
Source: Biol Reprod. 2020 Apr 25;103(2):254–63. doi: 10.1093/biolre/ioaa060 (PMC7401031; doi:10.1093/biolre/ioaa060)
Supplement: supplementary_figures_ioaa060 [file supplementary_figures_ioaa060.pdf]

Figure S1

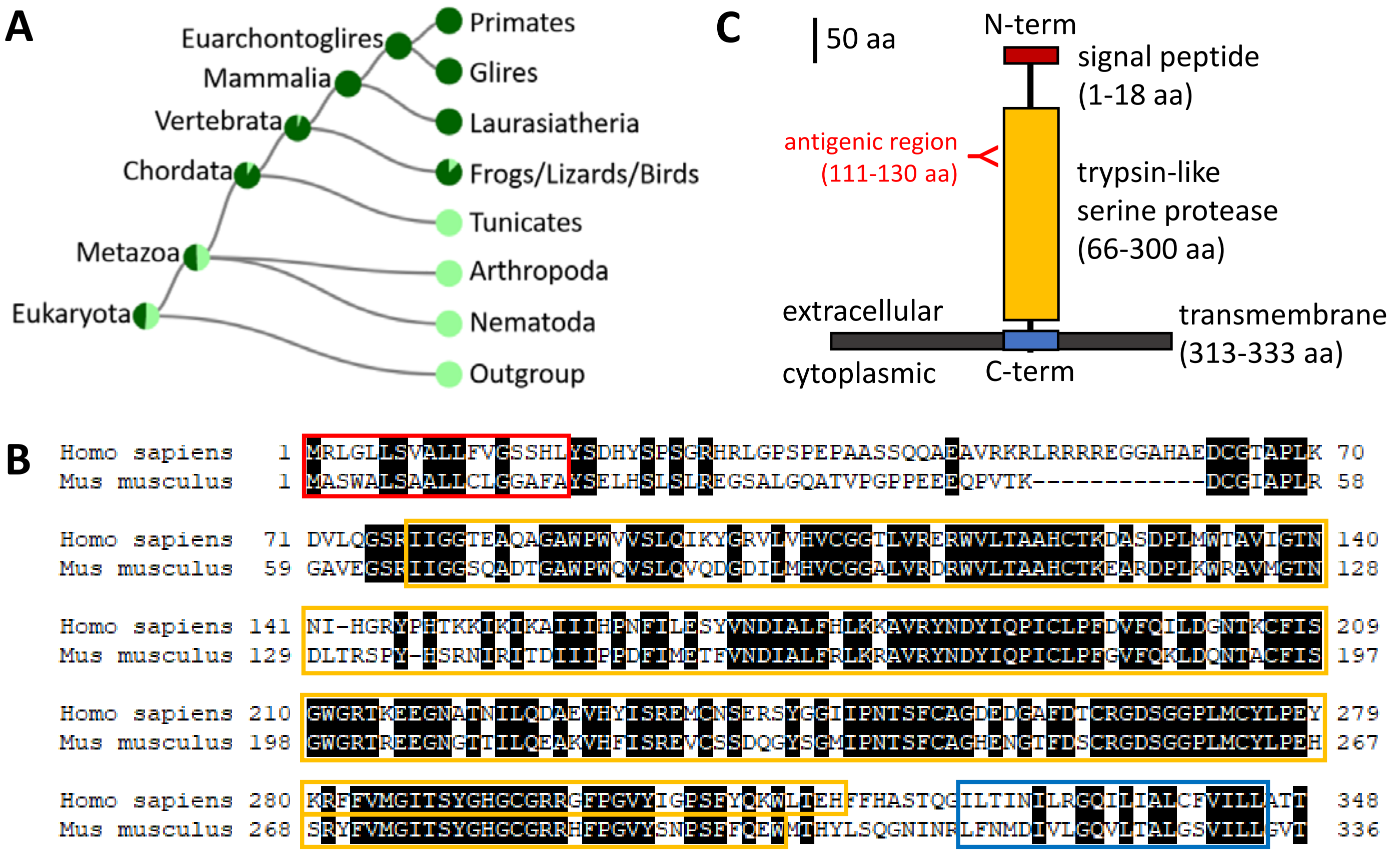
A
Euarchontoglires
Mammalia
Vertebrata
Chordata
Metazoa
Eukaryota
Primates
Glires
Laurasiatheria
Frogs/Lizards/Birds
Tunicates
Arthropoda
Nematoda
Outgroup
C
50 aa
N-term
signal peptide
(1-18 aa)
antigenic region
(111-130 aa)
trypsin-like
serine protease
(66-300 aa)
extracellular
cytoplasmic
C-term
transmembrane
(313-333 aa)
B
Homo sapiens 1 MRLGLLSVALLFVGSSHLYSDHYSPSGRHRLGPSPEPAASSQQAEAVRKRLRRRREGGAHAEDCGTAPLK 70
Mus musculus 1 MASWALSAALLCLGGAFAYSELHSLSLREGSALGQATVPGPPEEEQPVTK------------DCGIAPLR 58
Homo sapiens 71 DVLQGSRIIGGTEAQAGAWPWVVSLQIKYGRVLVHVCGGTLVRERWVLTAAHCTKDASDPLMWTAVIGTN 140
Mus musculus 59 GAVEGSRIIGGSQADTGAWPWQVSLQVQDGDILMHVCGGALVRDRWVLTAAHCTKEARDPLKWRAVMGTN 128
Homo sapiens 141 NI-HGRYPHTKKIKIKAIIIHPNFILESYVNDIALFHLKKAVRYNDYIQPICLPFDVFQILDGNTKCFIS 209
Mus musculus 129 DLTRSPY-HSRNIRITDIIIPPDFIMETFVNDIALFRLKRAVRYNDYIQPICLPFGVFQKLDQNTACFIS 197
Homo sapiens 210 GWGRTKEEGNATNILQDAEVHYISREMCNSERSYGGIIPNTSFCAGDEDGAFDTCRGDSGGPLMCYLPEY 279
Mus musculus 198 GWGRTREEGNGTTILQEAKVHFISREVCSSDQGYSGMIPNTSFCAGHENGTFDSCRGDSGGPLMCYLPEH 267
Homo sapiens 280 KRFFVMGITSYGHGCGRRGFPGVYIGPSFYQKWLTEHFFHASTQGILTINILRGQILIALCFVILLATT 348
Mus musculus 268 SRYFVMGITSYGHGCGRRHFPGVYSNPSFFQEWMTHYLSQGNINRLFNMDIVLGQVLTALGSVILLGVT 336

Figure S2

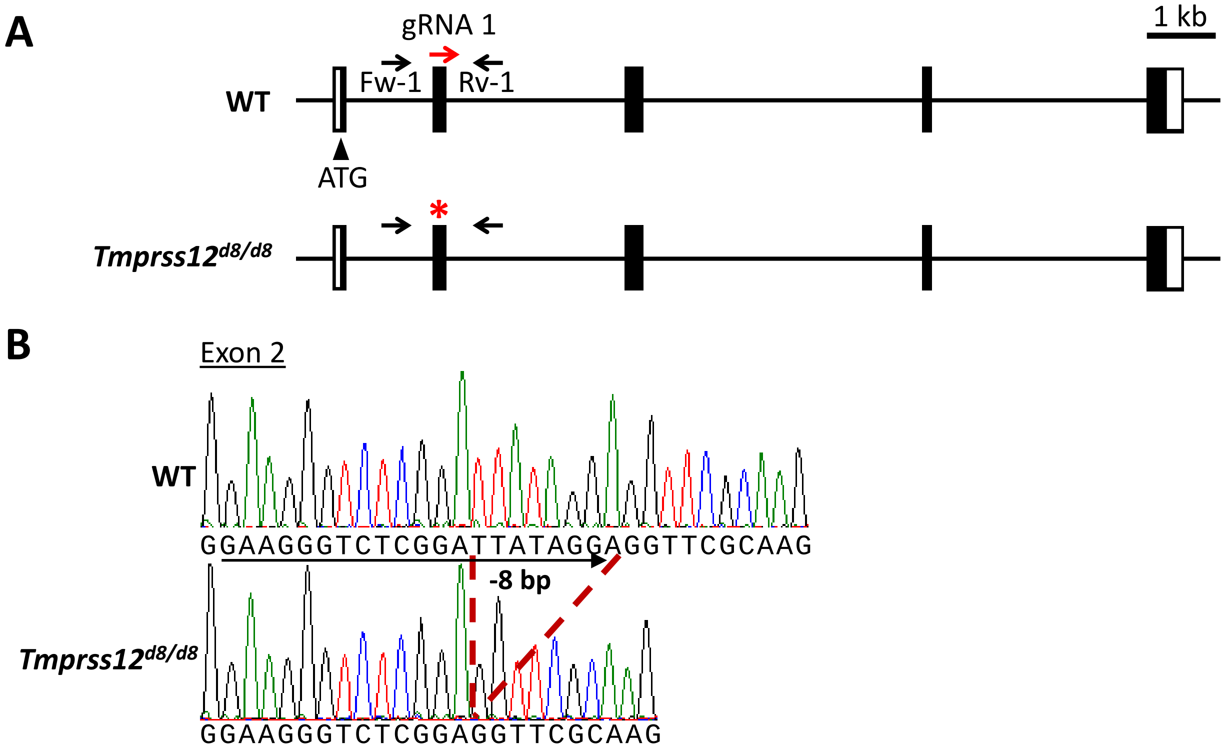

C

	1	63	70	336
**WT**	MASW---GSRIIGGS---LGVT			
Tmprss12 $^{d8/d8}$	MASW---GSRRFAS*			

Figure S3

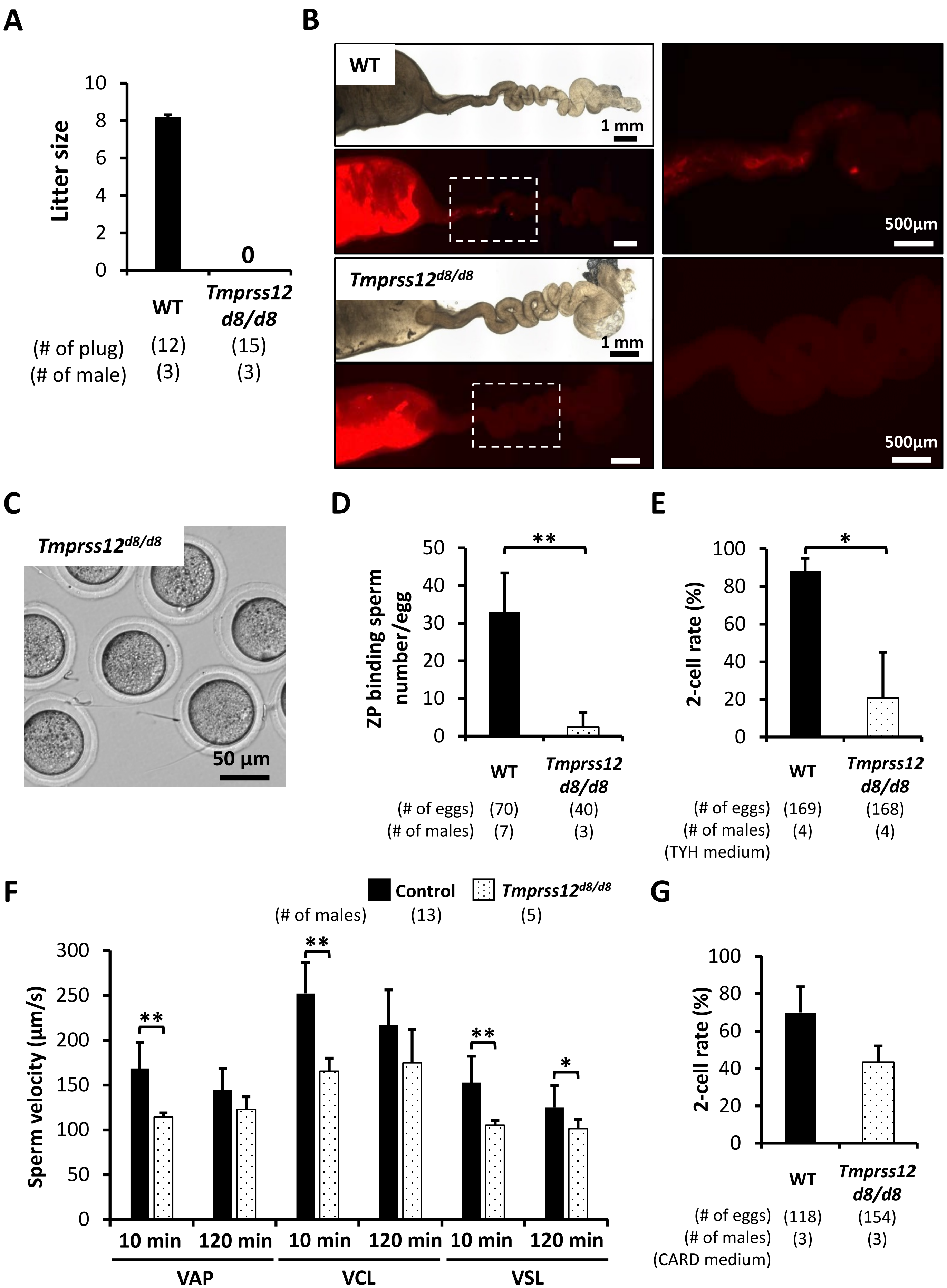
A
10
8
6
4
2
0
Litter size
0
WT
Tmprss12 d8/d8
(# of plug) (12) (15)
(# of male) (3) (3)
B
WT
1 mm
500µm
Tmprss12d8/d8
1 mm
500µm
C
Tmprss12d8/d8
50 µm
D
50
40
30
20
10
0
ZP binding sperm number/egg
**
WT
Tmprss12 d8/d8
(# of eggs) (70) (40)
(# of males) (7) (3)
E
100
80
60
40
20
0
2-cell rate (%)
*
WT
Tmprss12 d8/d8
(# of eggs) (169) (168)
(# of males) (4) (4)
(TYH medium)
F
Control
Tmprss12d8/d8
(# of males) (13) (5)
300
250
200
150
100
50
0
Sperm velocity (µm/s)
**
**
**
*
10 min
120 min
10 min
120 min
10 min
120 min
VAP
VCL
VSL
G
100
80
60
40
20
0
2-cell rate (%)
WT
Tmprss12 d8/d8
(# of eggs) (118) (154)
(# of males) (3) (3)
(CARD medium)

Figure S4

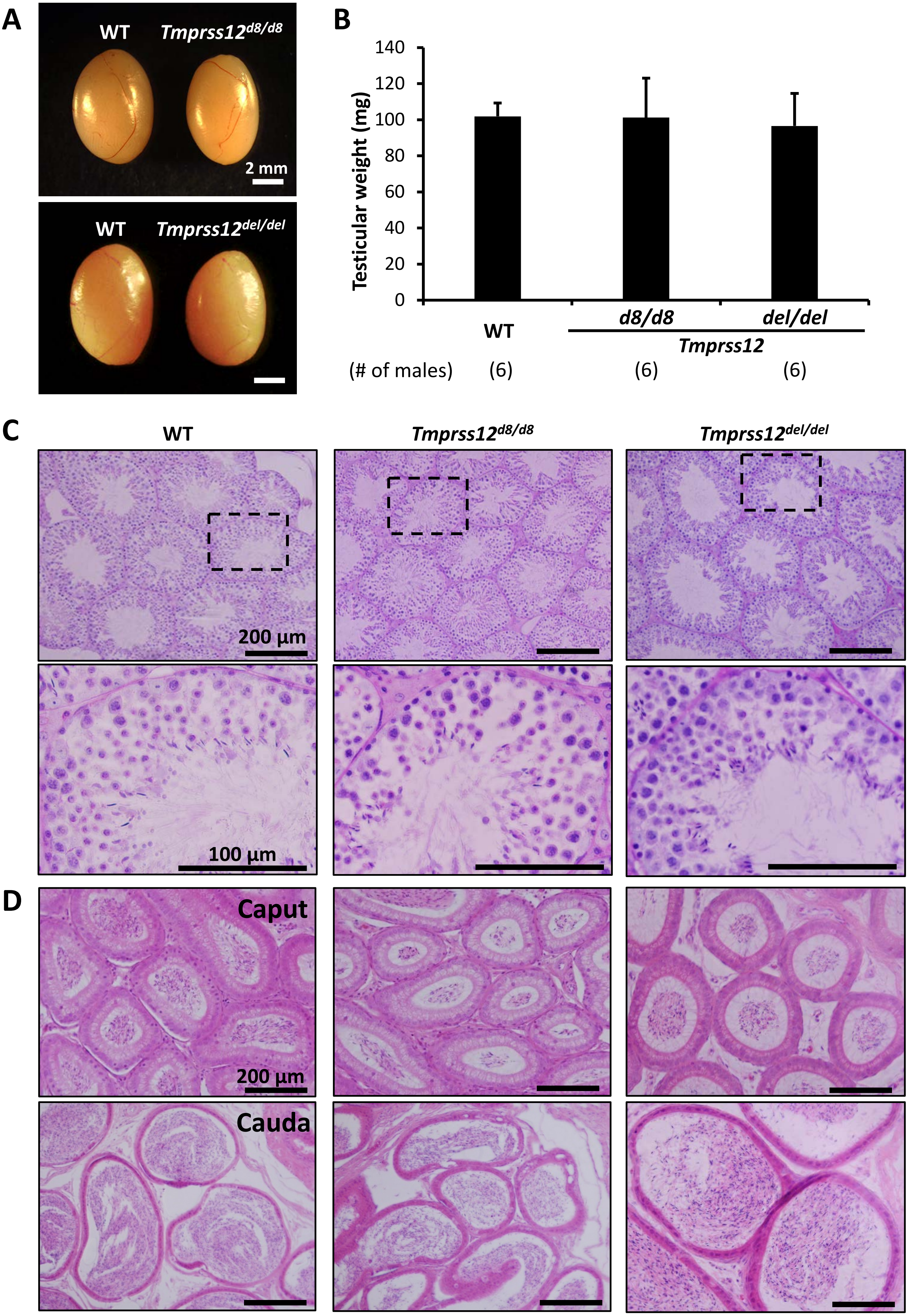
A
WT
Tmprss12d8/d8
2 mm
WT
Tmprss12del/del
B
Testicular weight (mg)
140
120
100
80
60
40
20
0
WT
d8/d8
del/del
Tmprss12
(# of males)
(6)
(6)
(6)
C
WT
Tmprss12d8/d8
Tmprss12del/del
200 µm
100 µm
D
Caput
200 µm
Cauda

Figure S5

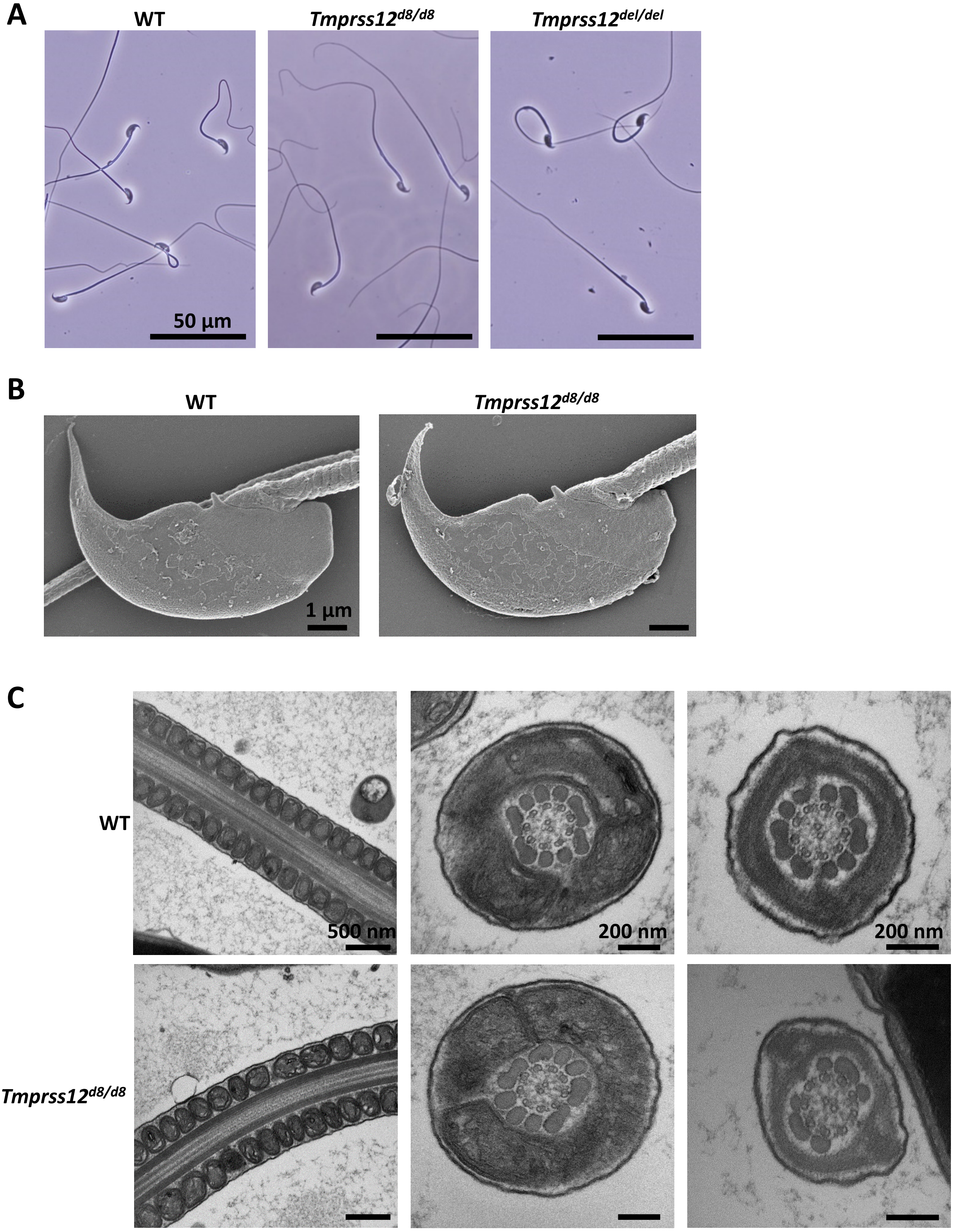
A
WT
Tmprss12d8/d8
Tmprss12del/del
50 µm
B
WT
Tmprss12d8/d8
1 µm
C
WT
Tmprss12d8/d8
500 nm
200 nm
200 nm
